# Injectable Decellularized Extracellular Matrix-Based Bio-Ink with Excellent Biocompatibility for Scarless Urethra Repair

**DOI:** 10.3390/gels9110913

**Published:** 2023-11-17

**Authors:** Wenzhuo Fang, Ming Yang, Yangwang Jin, Kaile Zhang, Ying Wang, Meng Liu, Yuhui Wang, Ranxing Yang, Qiang Fu

**Affiliations:** Department of Urology, Affiliated Sixth People’s Hospital, Shanghai Jiaotong University, No. 600 Yi-Shan Road, Shanghai 200233, China; fwz17794608029@163.com (W.F.); yangminguro@163.com (M.Y.); jinyw_med@163.com (Y.J.); great_z0313@126.com (K.Z.); sdzbbswangying@alumni.sjtu.edu.cn (Y.W.); yagmlight@163.com (M.L.); 18917328283@163.com (Y.W.)

**Keywords:** hydrogel, gelatin, dECM, scarless repair, tissue engineering

## Abstract

In recent years, decellularized extracellular matrices (dECM) derived from organs have attracted much attention from researchers due to their excellent biocompatibility, capacity to promote cell proliferation and migration, as well as pro-vascularization. However, their inferior mechanical properties, slow cross-linking, weak strengths, and poor supporting properties remain their inevitable challenges. In our study, we fabricated a novel dECM hydrogel with better crosslinking strength and speed, stronger support properties, and better mechanical properties. The hydrogel, which we named gelatin-based dECM powder hydrogel (gelatin-dECM hydrogel), was prepared by mixing dECM microparticles in gelatin solution and adding certain amount of 1-Ethyl-3-(3-dimethyl-aminopropyl-1-carbodiimide) (EDC) and N-hydroxysuccinimide (NHS). We evaluated the utility of this hydrogel by assessing the degradation rate, mechanical properties, and biocompatibility. The results showed that the gelatin-dECM hydrogel has high mechanical properties and biocompatibility and also has the ability to promote cell proliferation and migration. After injection of this hydrogel around the surgical sites of urethras in rabbits, the incorporation of dECM powder was demonstrated to promote angiogenesis as well as scarless repair by histological sections after surgery. The application of this novel hydrogel provides a new perspective for the treatment of post-traumatic urethral stricture.

## 1. Introduction

The treatment of post-traumatic urethral stricture remains one of the significant challenges in urology due to its large tissue defects, more severe trauma, scarce replacement tissue, and poor healing microenvironment [1,2]. Significantly, the above difficulties often contribute to inadequate vascularization of the repair, wound infection, increased inflammatory response, collagen deposition, and thus the formation of significant scar tissue, leading to eventual failure of the repair [3]. Although several therapeutic modalities such as natural oral mucosal grafts or flap grafts have been developed to replace the urethra for repair, they inevitably cause damage to the donor site and lead to a variety of complications [4]. Other multiple scaffolds in use today often have multiple drawbacks, such as adverse immune response leading to re-injury, more cumbersome preparation processes and higher costs, insufficient vascularization of the tissue after transplantation, etc. [5,6]. Therefore, it is imperative to prepare hydrogels that can be synthesized in a simple manner but have good biocompatibility and promote cell growth.

The extracellular matrix (ECM), as a network of macromolecules secreted by cells and located around the cells, is mainly composed of collagen, elastin, mucopolysaccharides, and glycosaminoglycans (GAG). It has been demonstrated to play a fundamental role in physiological and pathological processes such as cell proliferation, migration, inflammatory response, and angiogenesis [7,8,9]. Additionally, the extracellular matrix is mostly tissue-specific. From different tissues, the extracellular matrix usually has different types and contents of collagen, growth factors, and glycosaminoglycans. Due to its ability to mimic the initial microenvironment of different normal cells and tissues, it is often extensively exploited in tissue engineering and regenerative medicine [10,11]. For obtaining an extracellular matrix, researchers have devised many methodologies for decellularization. Decellularization is a novel technique in the field of tissue engineering research that disrupts cells into fragments, thereby selectively eliminating cells and maintaining an extracellular matrix. This method not only removes the cellular and DNA components that cause the immunogenic response but also preserves the biochemical structure, bioactivity, and mechanical integrity of the extracellular matrix, which allows bioactive components such as glycosaminoglycans, growth factors, and other growth factors to contribute to the promotion of cellular growth, reduction in inflammation, and vascularization [12,13].

Given these advantages, dECM hydrogels are increasingly used in regenerative medicine and tissue repair [14]. However, purely decellularized matrix hydrogels are limited in their applications due to their weak mechanical properties, poor supporting properties, low cross-linking strengths, and susceptibilities to degradation and inactivation by a harsh local microenvironment [15]. Encouragingly, gelatin, one of the most commonly used natural polymers for tissue repair and regeneration, is affordable and readily available, and its mechanical properties are concentration-dependent [16]. In addition, as a product of partial hydrolysis of collagen, it has low antigenicity, good biocompatibility, and specific cell adhesion motifs (RGD peptides); thus, it is commonly used for cell adhesion and growth and can be effectively assimilated in vivo without toxic degradation [17]. Although many studies have been reported on gelatin and organ dECM [18,19], there are fewer studies using bladder dECM in combination with gelatin; we are the first to apply it near the site of urethral trauma in order to achieve scarless urethral healing.

**Figure 1:**
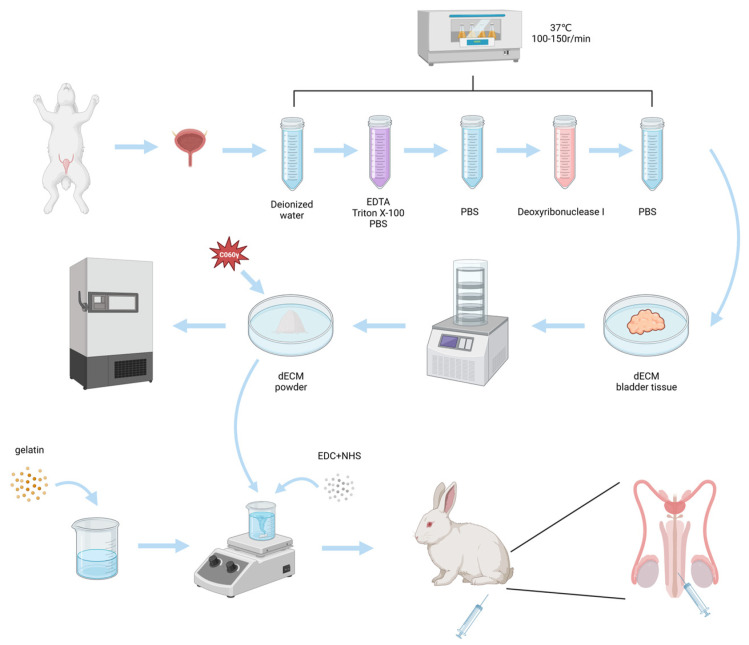
Schematic of the fabrication of gelatin-dECM hydrogel for urethra repair.

As shown in Figure 1, in this study, we synthesized a gelatin-dECM hydrogel using gelatin mixed with rabbit bladder dECM micro-particles and crosslinking agent EDC/NHS, which not only has definite mechanical strength and supportive property but also can fulfill the biological activity of dECM with the degradation of gelatin. In this study, as both gelatin and dECM are mainly proteins and contain a large number of carboxyl and amino groups, EDC/NHS can activate the carboxyl groups of both to bind with the amino groups of both, resulting in the formation of gelatin-dECM hydrogel. We evaluated the physical properties of the hydrogels by rheology and degradation rate measurements and the characteristic chemical bonding of the hydrogels by Fourier Transform Infrared Spectroscopy (FTIR) and X-Ray Photoelectron Spectroscopy (XPS). Finally, the repair results of the hydrogel were evaluated by histological staining of urethral sections and immunofluorescence staining.

## 2. Results

### 2.1. Characterization of Gelatin-dECM Hydrogel

**Figure 2:**
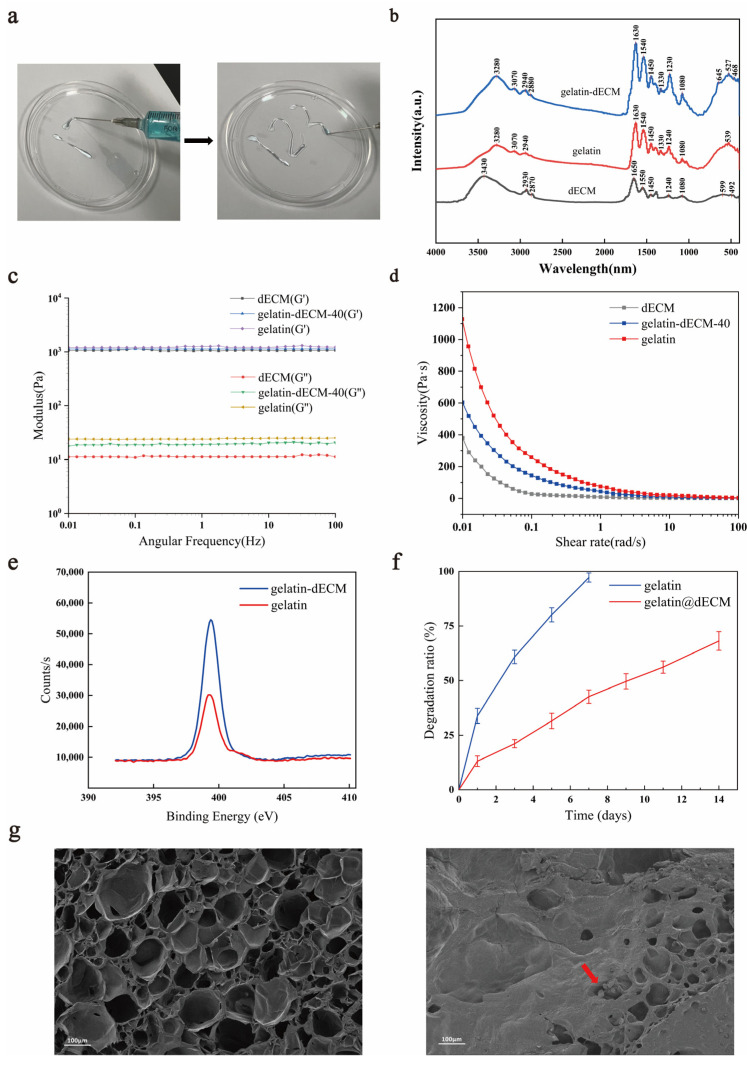
(**a**) Injection schematic and general view of injectable hydrogel. (**b**) Fourier Transform Infrared (FTIR) spectra of gelatin, dECM, and gelatin-dECM composite hydrogels. (**c**) The storage modulus (G′) and loss modulus (G″) of hydrogels at a fixed strain amplitude of 0.5% and a varying angular frequency (0.01∼100 Hz). (**d**) The viscosity of hydrogels by angular frequency sweep from 0.01 to 100 rad/s at 37 °C. (**e**) Gelatin-dECM characterization by XPS. Representative N1s scans of the gelatin sample (red) and gelatin-dECM sample (blue). (**f**) Degradation rates of above two scaffolds in phosphate buffer solution (PBS). (**g**) Vertical scanning electron microscopy (SEM) images of gelatin (**left**) and gelatin-dECM (**right**) hydrogels.

We prepared gelatin-dECM hydrogel by mixing gelatin, dECM, and EDC/NHS and activated the carboxyl group to combine it with the amino group to form an amide bond. The synthesized hydrogel was injectable, as shown in Figure 2a. Fourier Transform Infrared (FTIR) is one of the commonly utilized instruments for the detection of functional groups in compounds. It can utilize the vibrational absorption that occurs in chemical bonds or functional groups in molecules, and, depending on the different absorption frequencies, it shows different positions in the infrared spectrum, so that information about what kind of chemical bonds or functional groups are contained in the molecules can be obtained. The curve of the gelatin-dECM hydrogel contains the functional groups of both the gelatin and the dECM curves, which confirms the synthesis of the gelatin-dECM hydrogel synthesis (Figure 2b). The incorporation of gelatin enhanced the mechanical strength of the dECM hydrogel. To verify this, the rheological properties of the hydrogels were measured by measuring the storage modulus (G′) and loss modulus (G″) of the hydrogels at a fixed 0.5% strain amplitude. As shown in Figure 2c, the G′ values of the samples were consistently one to two orders of magnitude higher than the G″ values over the entire frequency range (0.01∼100 Hz), which suggests that the hydrogels developed a three-dimensional network. Moreover, we also demonstrated that the viscosity of the hydrogel continued to decrease with a gradual increase in the shear rate, which indicated its good injection properties (Figure 2d). Furthermore, after analyzing the samples using XPS, the results showed that the admixture of dECM powder resulted in a significant increase in the XPS signal of the gelatin-dECM hydrogel N1s (Figure 2e). This stems from the amide bond established between dECM powder and gelatin as well as the amide group of dECM powder. The degradation rate test results showed that gelatin-only hydrogel degraded faster (97.19 ± 2.10% on day 7), whereas the gelatin-dECM hydrogel degraded more slowly (68.17 ± 4.27% on day 14) (Figure 2f). We speculate that this is due to the fact that the dECM powder reduces the degradation rate of the hydrogel, and this property also realizes the advantage of the slow release and slow action of the bioactive substances in the gelatin-dECM hydrogel. In order to explore the ultrastructure of the hydrogel, we took scanning electron microscopy (SEM) shots of the cross section of the lyophilized hydrogel (Figure 2g). We can see that the gelatin hydrogel structure is more sparsely structured and abundant in pore structure. While the gelatin-dECM hydrogel structure is denser and less porous, the granular dECM powder can be seen in the cross section.

### 2.2. Characterization of Bladder before and after Decellularization

**Figure 3:**
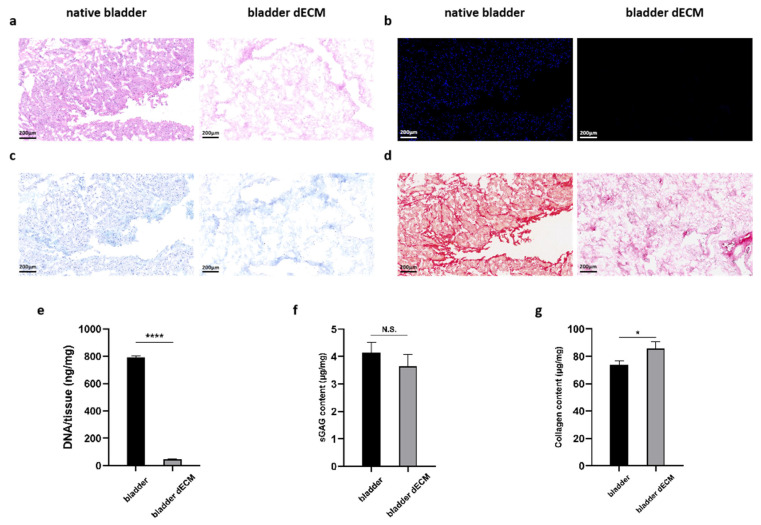
(**a**) DAPI and (**b**) H&E staining showed decellularization. (**c**) Alcian blue staining verified retention of glycosaminoglycans. (**d**) Sirius red staining demonstrated retention of collagen fibers. (**e**) Quantitative determination of DNA content, (**f**) sGAG retention, and (**g**) collagen preservation. Data are expressed as mean ± SD (*n* = 3). One-way ANOVA and Tukey’s multiple comparison test were used to indicate the significance, and “ns”: no significance. * *p* < 0.05, **** *p* < 0.0001.

Hematoxylin staining solution in (Hematoxylin eosin) HE staining is alkaline, which mainly causes the chromatin in the nucleus and nucleic acids in the cytoplasm to color violet-blue, whereas eosin is an acidic dye, which mainly causes the cytoplasm and components of the extracellular matrix to color red. HE staining is the most basic and widely used technique in histology and can be used to analyze the components and status of cells and extracellular matrix [20]. 2-(4-Amidinophenyl)-6-indolecarbamidine dihydrochloride (DAPI) is a fluorescent dye that binds to microbial DNA and often stains cellular DNA, mainly used for cellular localization and various cellular assays [21]. Sirius Red is a strongly acidic anionic dye that reacts with alkaline collagen and is mainly used for staining collagen and differentiating between different types of collagens [22]. Alcian blue is a cationic dye that binds to acidic groups (e.g., carboxyl groups and sulfate) to form an insoluble complex that stains acidic mucopolysaccharides in the cytoplasm blue and localizes them in the cytoplasm [23]. In this experiment, the HE staining of rabbit bladder ECM showed that the nuclear structure was effectively removed during decellularization without destroying the original ECM tissue components (Figure 3a). DAPI staining also confirmed the removal of nuclei (Figure 3b). In addition, we did Alcian blue and Sirius red staining, and they also confirmed that collagen and mucopolysaccharides were not destroyed during the decellularization process (Figure 3c,d). The decellularization process greatly reduced the amount of DNA in the tissues from 792.53 ± 9.40 ng/mg in native bladder tissue to 47.62 ± 3.85 ng/mg after decellularization (*p* < 0.0001; Figure 3e). The sGAG content of the decellularized bladder (3.65 ± 0.43 μg/mg) was similar to the level of the native bladder tissue (4.15 ± 0.35 μg/mg) (Figure 3f). Intriguingly, collagen quantification showed an increase in collagen content in the decellularized bladder (85.62 ± 5.14 μg/mg) compared to that of the native bladder tissue (73.63 ± 2.96 μg/mg) (Figure 3g), which may be attributed to the removal of cellular components that resulted in relatively higher collagen content. These results suggest that ECM components were well preserved after decellularization.

### 2.3. Assessment of the Ability to Promote Cell Proliferation and Growth

**Figure 4:**
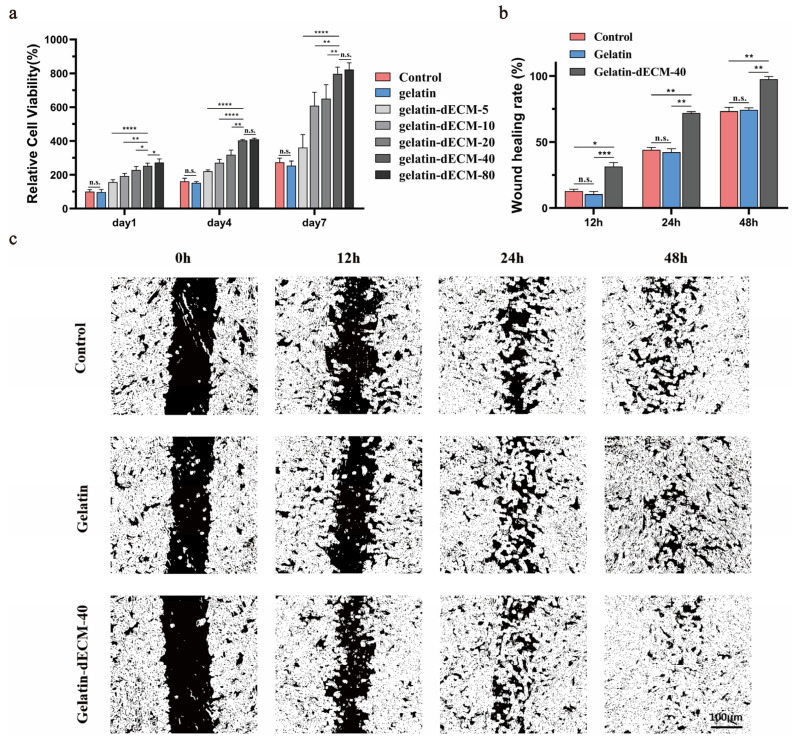
(**a**) The cell proliferation rates of ADSCs at 1, 4, and 7 days after treatment with various concentrations of dECM powder were detected by CCK-8. Data are expressed as mean ± SD (*n* = 6). Dark-field images (**c**) and statistics (**b**) of ADSCs cultured under diverse conditions (e.g., PBS, gelatin, and gelatin-dECM-40) in wound healing experiments, where the cell-free area was calculated by ImageJ 1.8.0 software, and laser irradiation was applied also. Data are expressed as mean ± SD (*n* = 3). One-way ANOVA and Tukey’s multiple comparison test were used to indicate the significance, and “ns”: no significance. * *p* < 0.05, ** *p* < 0.01, *** *p* < 0.001, **** *p* < 0.0001.

Given the well-established fact that organ decellularized matrices contain large amounts of growth factors and nutrients that can promote cell proliferation and migration [8], we determined the most appropriate dECM powder concentration by Cell Counting Kit-8 (CCK8) experiments. We found that the concentration of 40 mg/mL was the optimal concentration for adipose-derived stem cells (ADSCs) proliferation, as it showed a significant increase in proliferation rate relative to 5.10.20 mg/mL, whereas there was no statistically significant difference in the rate of increase in value for the powder concentration of 80 mg/mL (Figure 4a). In addition, this result also demonstrated that the gelatin hydrogel had excellent bio-safety, and its cellular activity was not statistically different from the control. Due to the consideration of the experimental cost, we chose the concentration of 40 mg/mL for the next experiments. Moreover, the cell scratch assay also confirmed the effect of dECM powder. Specifically, by semi-quantitative analysis of the coverage of different groups, ADSCs in the gelatin-dECM-40 group reached close to 100% coverage at 48 h, whereas the coverage of the other two groups was close to 70% (Figure 4b,c). These results demonstrated that the proliferation and migration of ADSCs were greatly enhanced with the stimulation of dECM.

### 2.4. In Vivo Scarless Regeneration of Urethral Wound in Rabbits

**Figure 5:**
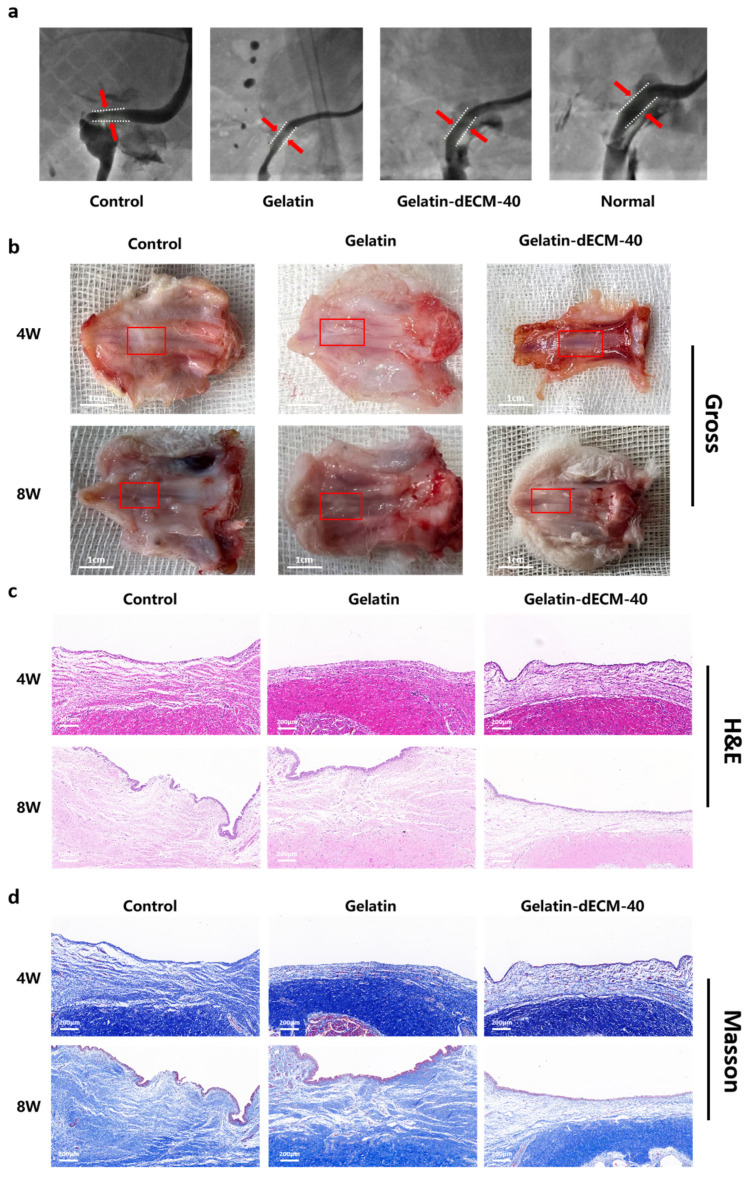
(**a**) Urethrographic image of the rabbit urethras in the normal group, control group (defect alone), gelatin group, and gelatin-dECM-40 group. (**b**) Digital photographs of in vivo tissue regeneration at the defect site after injection of different hydrogels. The red box in the figure indicates the site of repair after injury. Light microscopy images of H&E staining (**c**) and Masson’s staining (**d**) of in vivo tissue regeneration defects after the implantation of different scaffolds for pathological examination, after different post-processing and sectioning of engineered urethral tissues for gross appearance (scale bar = 1 cm) (**b**), H&E (**c**), and Masson’s (**d**) staining.

In experiments of urethral wound repair in vivo, we utilized a rabbit urethra model to characterize the ability of dECM to promote regeneration and healing. Urethrography is one of the best imaging techniques specifically designed to assess the repair and regeneration of urethral tissue, both in clinical and basic research. It was found that the urethra in both the control and gelatin groups had significant scarring, forming severe urethral strictures with poor urine passage (Figure 5a). In contrast, in the gelatin-dECM-40 group, the dorsal side of the urethra was smooth and flat with low urine blockage. The results in this group were approximately the same as the normal group, which indicated that dECM did have the effect of promoting urethral repair, reducing scar formation and lowering the blockage rate. The urethral tissue was sampled after 4 W and 8 W postoperatively. The results of the macroscopic view showed that scar tissues were formed in both the control and gelatin groups at 4 weeks postoperatively, and subsequently these scar tissues became more and more evident at 8 weeks, with enlargement and thickening (Figure 5b). In particular, for the gelatin-dECM-40 group, due to the ability of dECM to promote wound repair and regeneration as well as cell proliferation and migration, the urethral tissues were optimally restored, with no significant damage or scar tissue observed until 8 weeks postoperatively. In addition, the regenerated tissues were fully integrated with neighboring tissues, with no evident boundaries seen, and no evident inflammation or immune response was observed. The rabbits were also in a healthier overall state, which indicates that the scaffold has good biocompatibility.

### 2.5. Pathologic Examination of Urethral Repair

Pathological examination showed that the control and gelatin groups had the poorest healing wounds, with a large number of fibroblasts and collagen fibers accumulating near the wounds, forming a markedly thickened scar tissue and little vascular tissue. In addition, epithelial repair was also poorer at 28 days after surgery in the control and gelatin groups due to the destruction of some of epithelial cells by acute injury (Figure 5c,d). All these results showed that pure gelatin scaffolds had no effect on wound healing. In the gelatin-dECM-40 group, on the other hand, the tissue in the injured area was sparser and had continuity, with less distribution of collagen fibers. In addition, the inflammatory response in the injury area was low. Visible vascular tissue and epithelial cells could also be seen. After 8 weeks of injury, the repaired tissue was insignificantly different from normal urethral tissue. It can be seen that the addition of dECM promoted wound tissue regeneration as well as scarless healing of the urethra.

**Figure 6:**
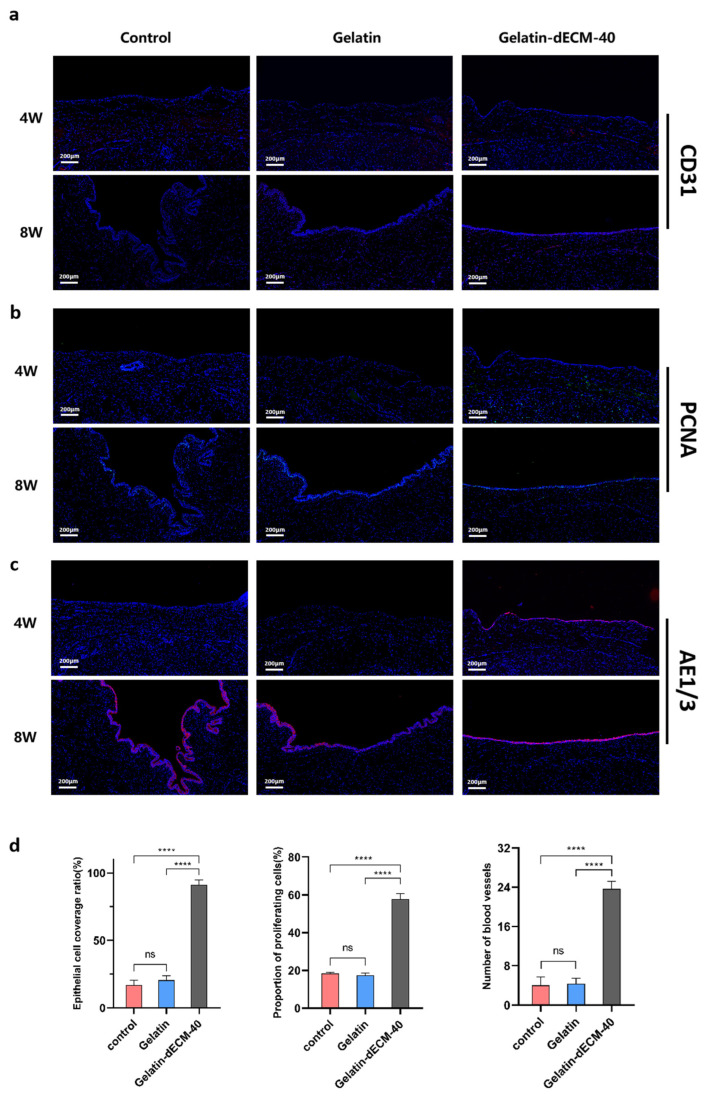
Immunofluorescence images of angiogenesis (CD31) (**a**), cell proliferation (PCNA) (**b**), and urethral epithelialization (AE1/3) (**c**), with blue representing the nuclei, and red and green representing the corresponding markers (image scale bars are: 200 µm). (**d**) Quantitative expression levels of AE1/AE3, PCNA, and CD31 in urethral sections for the assessment of epithelialization, cell proliferation, and angiogenesis at 4 weeks postoperatively under different treatment conditions (i.e., control, gelatin, and gelatin-dECM-40). Data are expressed as mean ± SD (*n* = 3). Significance was expressed using one-way ANOVA and Tukey’s post hoc test. **** *p* < 0.0001.

To have a more comprehensive understanding of the regeneration-promoting effects exerted by dECM, we experimented immunofluorescence staining for Platelet endothelial cell adhesion molecule-1 (PECAM-1/CD31) (Figure 6a), Proliferating cell nuclear antigen (PCNA) (Figure 6b), and Anti-pan Cytokeratin (AE1/AE3) (Figure 6c) to follow up to assess angiogenesis, epithelialization, and cell proliferation, respectively. The results showed thin epithelial cells 4 weeks postoperatively, fewer vascular endothelial cells, and insignificant cell proliferation in the control and gelatin groups at both 4 and 8 weeks postoperatively, all of which contributed to poor wound healing and eventual scar repair. Surprisingly, the epithelial cells in the gelatin-dECM-40 group could completely cover the wound, and the vascular endothelial cells and proliferating cells increased significantly. Our results after semi-quantitative analysis of immunofluorescence staining results showed that the CD31, AE1/AE3, and PCNA indexes were significantly improved in the dECM group compared with the control and gelatin groups (Figure 6d). This rapid epithelialization of wound healing, angiogenesis, and cell proliferation played a critical role in the regeneration of the eventual scarless repair.

## 3. Discussion

The treatment of urethral disorders such as urethral injuries and strictures has consistently been one of several challenges in urology. Currently, the first line of treatment often utilizes skin, oral mucosa, or intestinal autografts or xenografts to replace the defective tissues [24]. However, the use of autologous tissue for urethral reconstruction usually has the disadvantages of high surgical trauma, donor complications, and poor restoration of anatomical and physiological functions [25,26,27]. Furthermore, the limited availability of donor materials has not been overcome. Consequently, the current therapeutic paradigm of “repairing diseased tissue at the expense of healthy tissue” has been severely challenged [4,28]. Excitingly, the emergence of tissue engineering technology has now opened up new therapeutic ideas for urethral repair and reconstruction. Tissue engineering is a profoundly promising technology, and, as a rapidly growing field, it focuses on the use of biomaterials to repair damaged sites in vivo without causing an excessive immune response and can be mass-produced without damaging the donor site [29]. The use of tissue engineering for urethral repair has a very promising clinical application.

Gelatin, as a product of incomplete denaturation of collagen, is one of the most commonly used polymers for tissue engineering and has been used in a wide variety of applications due to its easy availability and inexpensive features. It has been acknowledged for its low antigenicity and low immunogenicity and has been approved by the Food and Drug Administration (FDA) as a medicinal ingredient for a long time [30]. Regrettably, although it can be degraded in vivo without toxicity, the disadvantage of its low bioactivity and lack of biological function cannot be ignored. In contrast, the extracellular matrix, as a network structure composed of cell-secreted proteins and polysaccharides, is abundant in a variety of growth factors and nutrients [31] and can facilitate tissue regeneration as well as cell proliferation, among other things. Inevitably, dECM hydrogel alone has low cross-linking strengths, poor mechanical properties, and is susceptible to denaturation by the harsh microenvironment of urothelial tissues, which results in the inability to maximize biological functions. Therefore, we combined both of them and formed gelatin-dECM hydrogel by cross-linking with EDC/NHS as cross-linking agent. The addition of dECM greatly enhanced the biological activity of the hydrogel and accomplished the function of promoting tissue repair and cell proliferation compared with that of gelatin hydrogel alone. While the incorporation of gelatin has greatly improved the mechanical strength and cross-linking strength of dECM hydrogels. In addition, gelatin as a carrier can block the invasion of some microenvironmental substances, which can protect dECM and make it function gradually with the degradation of gelatin.

With the accelerated development of tissue engineering technology, decellularized matrices from organs (e.g., heart, skin, cartilage, and liver, etc. [13,15,32,33]) have been increasingly used in the field of repair and regeneration. The decellularized matrix of bladder tissue used in this experiment has also been approved by the FDA as one of the most common dECM biomaterials for bio-regeneration since a long time ago [34,35]. Like dECMs of other organs, bladder dECMs contain a variety of growth factors, adhesion molecules, fibronectin, and cohesion modifiers, and other biologically active factors that can stimulate cell adhesion, tissue regeneration remodeling, and vascularization [36]. We have validated the capability of bladder dECM to promote cell proliferation and migration by CCK8 and scratch assays, as well as its ability to promote tissue regeneration and vascularization by injecting the hydrogel near the local urethral wound. We speculate that these excellent results are all attributable to the multiple bioactive factors enriched within bladder dECM, and the active substances in these ECMs play crucial roles in scarless urethral repair. However, its one unavoidable drawback, as with dECMs of other organ tissues, is that there are fewer donors, and they cannot be mass-produced, although the immunogenicity of the tissue is removed during the decellularization process. Therefore, our future research direction will focus on finding dECMs that can be substituted for other tissues of human organs, which can satisfy the advantages of dECMs that are rich in highly bioactive factors and solve the problem of donors to realize mass production, so as to lay a solid foundation for the development of dECM therapies that can eventually be applied in a clinic.

## 4. Conclusions

In our study, we presented the formulation of gelatin-dECM hydrogel, an injectable biologically active hydrogel rich in biological activity. It not only compensated for the lack of bioactivity of gelatin hydrogel but also improved the poor mechanical properties of the dECM hydrogel. The results demonstrated the ability of gelatin-dECM hydrogel to promote urethral scarless repair and regeneration as well as cell proliferation. However, there are some places for improvement in this experiment, for example, the bladder decellularized matrix used strictly speaking also requires the sacrifice of some of the organ tissues, which is not conducive to the mass production as well as the transformation of the hydrogel. In addition, the urethral environment is prone to bacterial growth, and if some kind of antimicrobial component could be added to the hydrogel, it would make the hydrogel more attractive. Overall, the gelatin-dECM hydrogel provides a new strategy for the development of post-traumatic urethral stricture therapies, which can be improved upon in the future to increase the likelihood of clinical translation and mass production.

## 5. Materials and Methods

### 5.1. Decellularization and Hydrogel Preparation

#### 5.1.1. Decellularization of Rabbit Bladder and Preparation of dECM Micro-Particles

Rabbit bladder sampling was obtained from normal adult male rabbits (6–8 months old/2.5–4 kg weight). Rabbit bladder tissues were washed with PBS buffer, and then the samples were immersed in decellularization solution and shaken on a shaker at 37 °C for 48 h at a frequency of 100–150 r/min. The specific decellularization solution was 10 mM Tris-HCl (Sigma, Shanghai, China), 10 mM EDTA (Sigma, Shanghai, China), 1% TritonX-100 (Sigma, Shanghai, China), and 1% SDS (Sigma, Shanghai, China), which was dissolved in deionized water with a pH value of 7.8. After the above-mentioned treatments, the cells were placed in PBS buffer and rinsed by shaking for 24 h with a shaking frequency of 100–150 r/min to remove residual cells and detergent. Subsequently, the tissues were placed in PBS buffer with deoxyribonuclease I (400 µg/mL, D5319, Sigma, Shanghai, China) and shaken for 24 h at a frequency of 100–150 r/min. Finally, the samples were rinsed again in PBS buffer with shaking. The decellularized samples were lyophilized and ground into micro-particles using a grinder and subsequently sterilized by C060 γ-ray irradiation. The finished products obtained were stored at −80 °C.

#### 5.1.2. Gelatin-Based Decellularized Extracellular Matrix Powder Hydrogel Preparation

Gelatin-dECM hydrogel was fabricated by mixing dECM powder into gelatin solution and adding cross-linker. In brief, gelatin (Sigma) (2.5%, MW = 219.66 Da) was added in deionized water and gently rotated for 30 min at 50 °C. The prepared gelatin mixture was sterilized using a filter (0.45 μm, Millipore, Danvers, MA, USA), and a portion of dECM powder was subsequently added to the gelatin solution and stirred well. Finally, Gelatin-dECM hydrogel was produced by adding EDC/NHS crosslinker. EDC is a mild cross-linking agent that acts as a carboxylate reactive reagent, activating the carboxyl group in biomolecules to react to form an amine-responsive O-acyl isourea intermediate, which rapidly reacts with the amino group to form an amide bond, releasing an isourea side product without causing protein denaturation [37]. While its intermediates are unstable in aqueous solutions, the two-step coupling procedure relies on NHS for stabilization. Furthermore, the presence of NHS can increase the efficiency of EDC-mediated coupling by several fold [38]. The use of EDC/NHS as a cross-linking agent to cross-link to form a hydrogel is usually sufficient at room temperature, with a cross-linking time of 3–5 min. The specific concentrations and the weight ratios of each component are shown in Table 1.

### 5.2. Characterization of Gelatin-dECM Hydrogel

#### 5.2.1. Fourier Transform Infrared (FTIR)

The FTIR spectra of gelatin, dECM and gelatin-dECM hydrogels were studied under IR irradiation at different wavelengths (from 4000 to 400 nm) using a Nicolet-iS20 spectrometer (Thermo Scientific, 81 Wyman Street, Waltham, MA, USA) at room temperature.

#### 5.2.2. X-ray Photoelectron Spectroscopy (XPS)

We performed XPS measurements of gelatin, dECM, and gelatin-dECM hydrogels for peptide conjugation assessment using an ESCALAB QXi XPS spectrometer (Thermo Fisher Scientific, 81 Wyman Street, Waltham, MA, USA). For analysis, hydrogels were lyophilized, and powdered compacts were laid flat on conductive carbon tape. The samples were analyzed using micro-focused radiation with a spot size of 400 μm, and fine spectral tests were performed on the C and N orbitals.

#### 5.2.3. Rheological Measurement of the Gelatin-dECM Hydrogel

The rheological properties of gelatin, dECM, and gelatin-dECM hydrogels were characterized using a 15 mm parallel plate rheometer (HAAKE MARS60, MASTER ESCH 7,248,691, Vreden, Germany). Hydrogels were subjected to a frequency sweep ranging from 0.01 to 100 Hz at a constant amplitude (γ = 0.5%). During the test, the temperature of sample stage was set at 37 °C. The changes in storage modulus (G′) and loss modulus (G″) with angular frequency were recorded. The injectability of hydrogels were measured by the changes in viscosity with shear rate. Shear rates ranged from 0.01 s^−1^ to 100 s^−1^.

#### 5.2.4. Scanning Electron Microscopy Analysis of Gelatin-dECM Hydrogels

Scanning electron microscopy (SEM; ZEISS Sigma 300, Oberkochen, Germany) was used to measure the morphologies and pore sizes of gelatin, dEC, and gelatin-dECM hydrogels. Hydrogels were lyophilized overnight under vacuum and then pre-coated with a gold layer on the support using a sputter coater for 45 s to make the surface conductive before imaging. For the purpose of cross-sectional analyzation, the samples were cut down the middle. At last, the samples were imaged on a scanning electron microscope using an accelerating voltage of 3 kV and a secondary electron detector.

#### 5.2.5. Degradation Assay

We measured the degradation characteristics of gelatin and gelatin-dECM hydrogels. At the beginning of the experiment, we chose two groups of hydrogels of the same quality, with three samples in each group. The hydrogels were placed in 3 mL of PBS buffer, and the degradation process was carried out in a shaker at 37 °C and 100 rpm. At the end of shaking, the samples were collected and lyophilized, and then their weights were measured separately. The degradation rate was calculated according to Equation

$$D=\frac{\left(Wo-Wt\right)}{Wo}\times 100\%$$

Note, W_t_ is the mass of residual hydrogel after 1, 3, 5, 7, 10, and 14 days of degradation, and W_0_ is the initial mass of the hydrogel.

### 5.3. Characterization of Rabbit Bladder dECM

#### 5.3.1. DNA Assay

The degree of decellularization of bladder tissue was assessed by DNA quantification. After extracting DNA from rabbit bladder and dECM by Genomic DNA Extraction Kit (Servicebio, Wuhan, China), the extracted genomic DNA was measured using Nanodrop 2000 (Thermo Scientific, 81 Wyman Street, Waltham, MA, USA).

#### 5.3.2. Sulfated Glycosaminoglycans and Total Collagen Assay

To assess the composition of ECM, bladder tissue and bladder dECM were assayed for total collagen and sulfated glycosaminoglycans (sGAG). The sGAG were assayed using the Blyscan sGAG Assay Kit (Biocolor, Carrickfergus, UK). Total collagen was measured using the SircolTM Collagen Assay Kit (Biocolor, Carrickfergus, UK). Specific assay procedures were performed according to the manufacturer’s instructions.

#### 5.3.3. Histological and Fluorescence Staining Analysis

Fresh bladder tissue and bladder dECM were fixed with 4% neutral paraformaldehyde overnight at 4 °C, subsequently dehydrated and embedded in paraffin. Samples were cut into 6 µm thick sections using a microtome. Sections were stained with hematoxylin and eosin (H&E) to observe the tissue condition and cellular distribution, stained with Alcian blue to observe the distribution of proteoglycans, and stained with Sirius red to observe the distribution of collagen. The sections were stained with 1 µg/mL 4′,6-diamidino-2-phenylindole (DAPI; Sigma, Shanghai, China) solution, followed by rinsing the slides with PBS and observing the distribution of nuclei in the tissues with a fluorescence microscope (DMi8; Leica, Wetzlar, Germany).

### 5.4. Cell Cytotoxicity and Proliferation Assessments

We immersed gelatin hydrogel in 35 mL of culture medium and shaken on a shaker at 37 °C for 24 h at a frequency of 100–150 r/min. The obtained leach liquor was filtered through a filter (0.45 μm) followed by addition of dECM powder to the leach liquor according to the concentrations of 5.10.20.40 and 80 mg/mL to finally fabricate the novel culture medium. The specific components of the novel medium and their concentrations in each well are shown in Table 2. The cytotoxicity and proliferation of the cells after 1, 3, and 7 days of incubation with novel culture medium were determined by CCK-8 assay kit (Servicebio, Wuhan, China). At each time point, the culture medium was removed, and the cells are incubated with 100 µL of fresh culture medium containing 10 µL CCK-8 at 37 °C for 3 h. The absorbance of the supernatant of each well was measured at 450 nm using a spectrophotometer, the values were recorded, and the survival rate was calculated compared to the control without any treatment.

### 5.5. Wound-Healing Experiment

ADSCs cells were manually scratched in six-well plates with a 200 µL specialized pipette tip. Subsequently, photographs were taken of the wound areas of the different groups with a light microscope (Olympus, Tokyo, Japan) after 0, 24, and 48 h of incubation in the novel medium described above, respectively. Cell-free areas were measured and analyzed by ImageJ 1.8.0 software.

### 5.6. In Vitro Extraction and Culturing of Primary Cells of ADSCs

Adipose tissue was collected from the groin of adult New Zealand rabbits, and the process was kept sterile. The collected tissues were rinsed 3 times each in PBS buffer and PBS buffer containing penicillin and streptomycin. The rinsed adipose tissue was thoroughly minced with sterile shears and digested with 1% collagenase (Nordmark, Hamburg, Germany) for 2 h. The collected cell suspension was inoculated onto petri dishes and incubated with Dulbecco’s Modified Eagle Medium (DMEM) medium containing 10% fetal bovine serum and 1% penicillin/streptomycin antibiotics in an incubator at 37 °C and 5% CO_2_ concentration.

### 5.7. In Vivo Modeling of Urethral Injury on Rabbits

Twenty-four male New Zealand rabbits (6–8 months of age/2.5–4 kg body weight) were divided into three groups: control, gelatin, and gelatin-dECM. The dorsal urethral injury model was used in this study. Not only due to the susceptibility of ventral wound healing after multilayered suturing of the urethral skin but also due to the fact that the dorsal side is more suitable for surgery, observational assessment, and sampling. The specific injury site was 1.5 cm from the external urethral opening, which helped to localize the tissue collection. The method of injury was to expose the rabbit urethra followed by clamping of the penile and urethral mucosa with hemostatic forceps to simulate a clinical urethral injury. Subsequently, hydrogel was injected around the area of injury, and, to eliminate the effect of injection, saline was injected in the control group. According to the grouping, rabbits were executed at 4 and 8 weeks, respectively. The urethral tissue at the site of injury was immersed in a 4% formalin solution and fixed for 24 h. After dehydration using graded ethanol, these tissues were embedded in paraffin and subsequently sectioned using a microtome, and the sections (4 µm) were examined histologically by HE and Masson staining.

### 5.8. Immunofluorescence Analysis

The expression of AE1/AE3, CD31, and PCNA in urethral sections from different groups of rabbits was analyzed by immunofluorescence staining. The pictures were captured on a phase-contrast microscope. Antigen repair: Sections were subjected to antigen repair in a repair cassette in EDTA antigen repair buffer (PH8.0) in a microwave oven. After 8 min of medium heat and 8 min of cease-fire, the slides were transferred to medium-low heat for 7 min. after natural cooling, the slides were placed in PBS and washed by shaking on a decolorizing shaker for 3 times, 5 min each time. Circle quenching of autofluorescence: after slightly drying the slices, draw a circle around the tissue with a histochemical pen, add autofluorescence quencher inside the circle for 5 min, and rinse with running water for 10 min. serum closure: Incubate for 30 min with a drop of Bovine Serum Albumin (BSA) inside the circle. Adding primary antibody: Gently shake off the sealing solution, add the primary antibody prepared by PBS in a certain proportion onto the slides, incubate the slides flatly at 4 °C in a wet box overnight. Add secondary antibody: the slides were washed in PBS on a decolorizing shaker for 3 times, each time for 5 min, the sections were shaken slightly and then a drop of primary antibody of the corresponding genus was added in a circle to cover the tissues, incubate for 50 min at room temperature, protected from light. DAPI re-staining of cell nuclei: the slides were washed in PBS (PH7.4) in a decolorizing shaker 3 times, each time for 5 min. The slides were shaken dry, the DAPI staining solution was added dropwise in a circle, followed by incubation for 10 min at room temperature, protected from light. The slides were washed three times in PBS on a decolorizing shaker for 5 min each time, and the sections were dried slightly and sealed with an anti-fluorescence quenching sealer and stored in a 4 °C light-proof slide box. Microscopic examination and photographing: the sections were observed under a fluorescence microscope, and the images were collected. (DAPI UV excitation wavelength 330–380 nm, emission wavelength 420 nm, blue light; CY3 excitation wavelength 510–560, emission wavelength 590 nm, red light, FITC excitation wavelength 465–495 nm, emission wavelength 515–555 nm, green light). Semi–quantitative analysis was performed using ImageJ 1.8.0 software.

### 5.9. Urethrography Analysis

Eight weeks after surgery, different groups of rabbits were subjected to urethrography before sacrifice. An 8 F catheter was inserted into the urethra up to the bladder, and radiographs were taken after injecting 20 mL of iodine contrast into the bladder. The degree of urethral stricture was assessed as the ratio of the width of the urethral stricture to the total width of the normal urethra.

### 5.10. Statistical Analysis

Data were obtained from at least three independent experiments, and all variables are expressed as mean ± standard deviation (SD). Differences between experimental groups were analyzed using one-way ANOVA and Tukey’s multiple comparison test. Single, double, triple, and quadruple asterisks represent *p* < 0.05, 0.01, 0.001, and 0.0001, respectively, and *p* < 0.05 was considered statistically significant. All statistical analyses were performed using SPSS 13.0 software (SPSS Inc., Chicago, IL, USA).

## Figures and Tables

**Table 1:** Concentrations of different substances in hydrogels as well as cross-linking agent concentrations.

Hydrogel	Gelatin (wt%)	dECM (wt%)	EDC (wt%)	NHS (wt%)	Final Concentration (wt%)
gelatin	6.5	/	2	2	6.5
dECM	/	6.5	2	2	6.5
Gelatin-dECM-40	2.5	4	2	2	6.5

**Table 2:** Different components in the novel culture medium for cytotoxicity and proliferation assessment and wound-healing experiment.

	Gelatin (wt%) in Leach Liquor	Volume of Liquid (mL)	Weights of dECM Powder (mg)	dECM Concentration of Novel Culture Medium (wt%)
control	0	5	0	0
gelatin	2.5	5	0	0
Gelatin-dECM-5	2.5	5	25	0.5%
Gelatin-dECM-10	2.5	5	50	1%
Gelatin-dECM-20	2.5	5	100	2%
Gelatin-dECM-40	2.5	5	200	4%
Gelatin-dECM-80	2.5	5	400	8%
Total		35	775	

## Data Availability

All data and materials are available on request from the corresponding author. The data are not publicly available due to ongoing research using a part of the data.
